# Isolated Superior Mesenteric Vein Tumor Thrombus in a Patient with Gastric Cancer

**DOI:** 10.1155/2018/3648436

**Published:** 2018-03-27

**Authors:** Barış Özcan, Metin Çevener, Ayşegül Kargı, Mustafa Özdoğan, Alihan Gürkan

**Affiliations:** ^1^Department of General Surgery, Medstar Antalya Hospital, Antalya, Turkey; ^2^Department of Radiology, Medstar Antalya Hospital, Antalya, Turkey; ^3^Department of Medical Oncology, Medstar Antalya Hospital, Antalya, Turkey

## Abstract

Tumor thrombus in the portal vein can rarely originate from gastric cancer via hematogenous spread, with only few case reports published in the literature. Isolated superior mesenteric vein tumor thrombus in gastric cancer has not been previously reported. A 61-year-old male patient who had undergone distal gastrectomy and gastroenterostomy for gastric ulcer 20 years ago was diagnosed with an obstructive tumor originating from the gastroenterostomy anastomosis site on upper gastrointestinal endoscopy that was performed for complaints of fatigue, oral feeding problems, and anemia. The PET-CT imaging revealed a hypermetabolic mass in the gastroenterostomy region along with hypermetabolic suspected tumor thrombus in the superior mesenteric vein (SMV). A suspected tumor thrombus with contrast enhancement that completely obstructed the SMV was detected on triphasic abdominal computed tomography. Decision for surgery was made due to gastric tumor obstruction. Firstly, lesions suspected with tumor thrombus were extirpated from the SMV and sent to frozen section. Then, it was completely recanalized. A locally advanced tumor originating from the gastroenterostomy anastomosis site that totally obliterated the lumen was observed on surgical exploration. After proving tumor thrombus by frozen, near-total gastrectomy was performed for palliative purposes. Histopathologic examination of the specimen showed gastric invasive adenocarcinoma and tumor thrombi in the SMV (T4N2M1). The patient received adjuvant chemotherapy, and he is at his 22nd-month follow-up with extensive hepatic metastases and intra-abdominal disease. It should be kept in mind that gastric cancer may lead to portal vein tumor thrombus or that it may rarely be associated with an isolated SMV tumor thrombus, both of which are associated with poor prognosis.

## 1. Introduction

Gastric cancer mainly spreads via lymphatic and hematogenous routes. In gastric cancer, tumor cells enter into the portal system by hematogenous route and lead to hepatic metastases. Nevertheless, tumor thrombi in the portal vein itself can rarely be detected in patients with gastric cancer [1]. Only few cases have been reported in the literature regarding portal vascular tumor thrombosis due to gastric cancer, which is considered as a poor prognostic factor, and the survival rate is reported to be quite low in these patients [2, 3].

In the literature, it has been shown that portal vein tumor thrombus in patients with gastric cancer may be associated with splenic vein, right and/or left gastric vein, or superior mesenteric vein (SMV) tumor thrombi [4]. Tumor thrombus development in these locations is attributed to gastric venous drainage pathways, while gastric cancer associated with isolated superior mesenteric vein tumor thrombus has not been previously reported in the literature.

In this case report, we aimed at presenting the diagnostic and therapeutic stages of an obstructive gastric cancer, which was diagnosed in a patient who had undergone partial gastrectomy for benign causes who then presented with gastric cancer in the gastrojejunostomy site along with isolated superior mesenteric vein tumor thrombus.

## 2. Case Report

**Figure 1:**
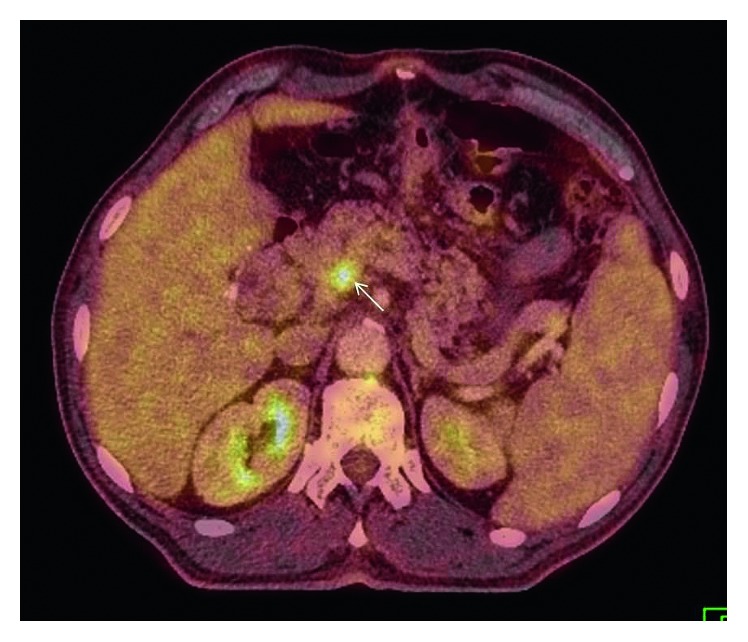
Fused axial image of 18-FDG-PET/CT scan shows 18-FDG uptake of thrombus in the superior mesenteric vein. This finding was considered as hypermetabolic tumor thrombus.

**Figure 2:**
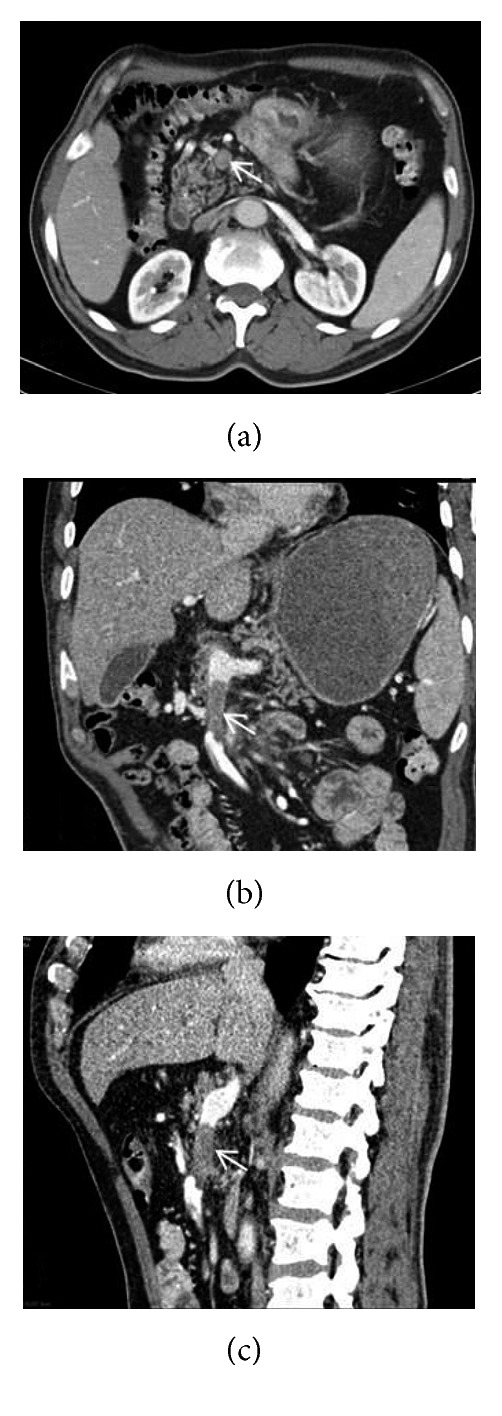
Axial (a), coronal (b), and sagittal (c) slices of abdominal CT show contrast media defect due to tumor thrombus (arrows) in the superior mesenteric vein.

A 61-year-old male patient who had undergone distal gastrectomy and gastroenterostomy due to gastric ulcer 20 years ago presented with new onset fatigue, oral feeding problems, and anemia. The upper gastrointestinal system endoscopy revealed an obstructing tumor at the gastroenterostomy site. Histopathologic examination of the endoscopic biopsy showed moderately differentiated adenocarcinoma. The PET-CT revealed a hypermetabolic mass in the gastric anastomosis site along with hypermetabolic activity in the superior mesenteric vein (SMV) suspected with tumor thrombus (Figure 1). A contrast-enhanced thrombus misgiving for tumor was detected within a 4 cm segment of the SMV proximal to the splenic confluence that completely obstructed the lumen on triphasic computed tomography (Figure 2). Mesenteric venous drainage was maintained through collateral veins that drained into the portal vein. Portal vein tumor involvement was not detected, and the portal vein was fully patent.

**Figure 3:**
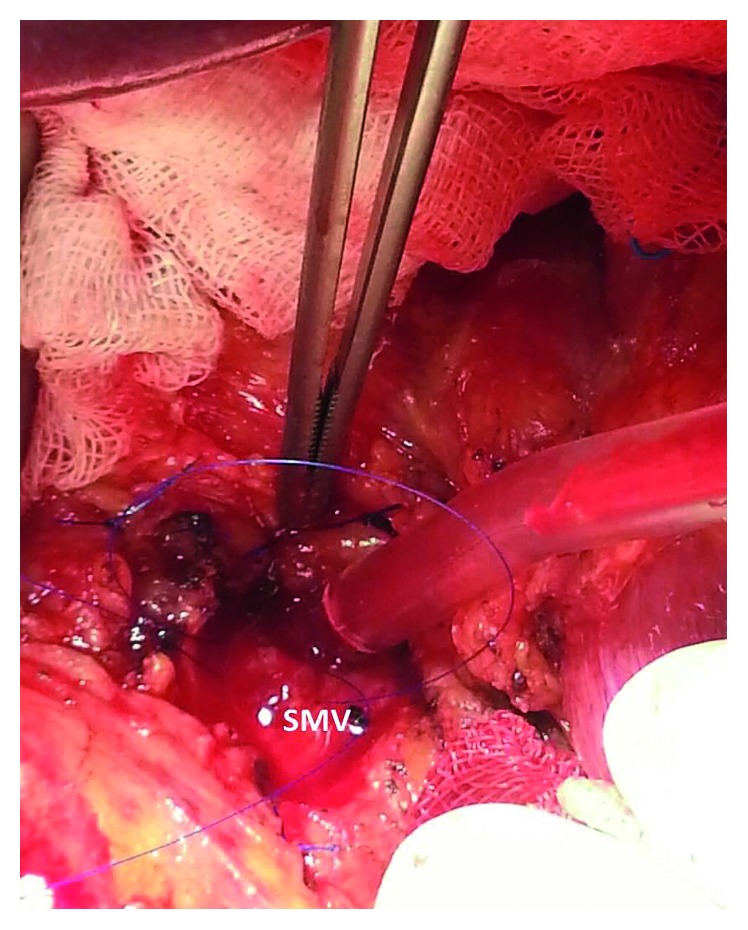
The SMV dissection near the splenic confluence.

**Figure 4:**
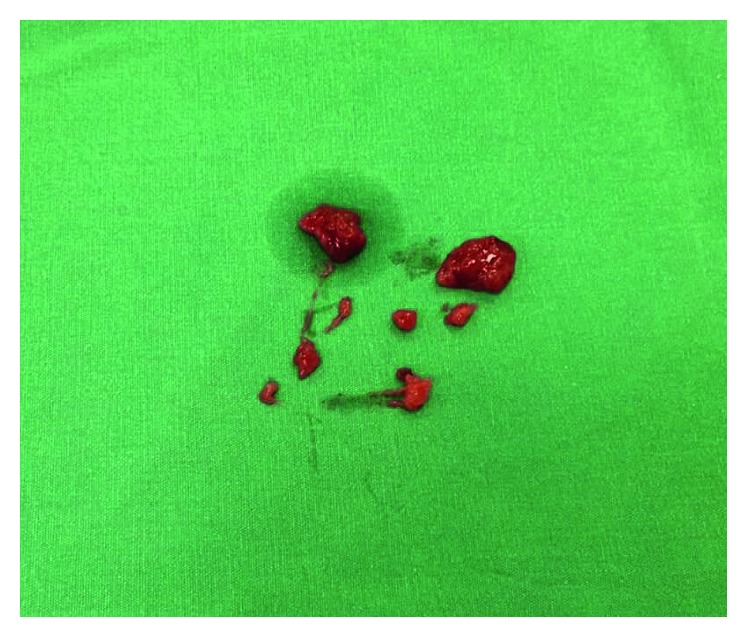
Extirpated tumor thrombus within the SMV.

Decision for surgery was made due to tumor obstruction. On surgical exploration, a tumor that originated from the gastroenterostomy anastomosis site with near-complete obstruction, infiltrating the surrounding tissues was observed. Firstly, to ensure that thrombus was tumor, the SMV was dissected and opened vertically near the splenic confluence under vascular control (Figure 3). The SMV was completely occluded with no blood flow. The thrombus was extirpated within the SMV by direct removal and by using a Fogarty catheter (Figure 4). Following recanalization of the SMV, reflow was allowed and the vein was closed with primary repair. The thrombus was sent to frozen section, and the result revealed tumor. Therefore, the patient was considered to be in the metastatic stage, but palliative surgery for gastric cancer was decided due to luminal obstruction. Gastric tumor tissue was completely dissected from the surrounding tissues followed by near-total gastrectomy and Roux-en-Y gastroenterostomy. The patient was discharged with low-molecular-weight heparin treatment without any problems in the intraoperative and postoperative period.

**Figure 5:**
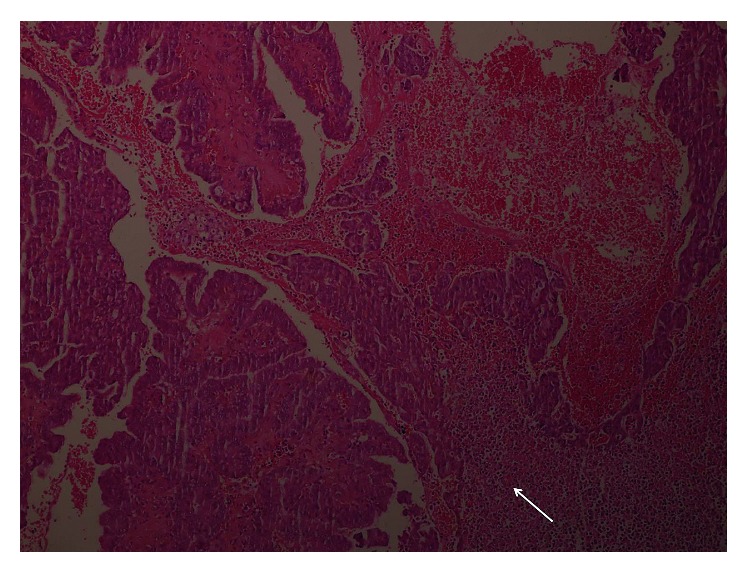
H&E (×100) shows tumor cells (arrow) in bloody background.

Histopathologic examination of the surgical specimen revealed gastric invasive adenocarcinoma. Infiltrated surrounding serosal fat planes by gastric tumor was detected (T4). Six surrounding lymph nodes from specimens were resulted as metastatic (N2). Lesions removed from the SMV have been reported to be tumor thrombi (T4N2M1) (Figure 5).

Postoperative abdominal computed tomography showed no evidence of thrombus in the SMV, but the SMV was obliterated and the drainage was still provided by collateral veins. The patient received 5 cycles of systemic paxlitaxel and carboplatin adjuvant chemotherapy. He is at his 22nd-month follow-up with extensive liver, peritoneal, and omental metastases.

## 3. Discussion

Gastric cancer spreads via lymphatic, hematogenous, and peritoneal routes and local invasion. Perigastric and paraaortic lymph nodes are involved first through lymphatic spread, whereas liver metastases develop via the portal venous system by hematogenous spread [5]. Although rare, portal vein tumor thrombi can also be detected in case of hematogenous dissemination [6–11].

Although gastric veins usually drain directly into the portal vein, they can also drain first into the superior mesenteric vein (SMV) and then the portal vein via the right and left gastroepiploic veins. Therefore, tumor cells may spread into the portal system in patients with gastric cancer to cause a tumor thrombus there or, rarely, to create a tumor thrombus within the SMV [1, 4].

Furui et al. reviewed published patients with gastric cancer in whom portal vein thrombosis was detected in the literature along with their own series in 1996 [4]. Isolated SMV tumor thrombus was not detected in any of the 15 patients presented in that review. It was reported that tumor thrombus in the SMV was usually accompanied by concurrent portal vein or right gastroepiploic vein tumor thrombus.

In our case, we thought that the gastric venous drainage route into the SMV was altered due to the previous distal gastrectomy, especially because of absence of the right gastroepiploic venous drainage. Since a tumor originating from the gastrojejunostomy site was detected in our case, it was concluded that venous drainage was obtained by jejunal mesenteric veins instead of gastric drainage routes, thus resulting in an isolated tumor thrombosis within the SMV. Similar to our case, Ishigami et al. [7] reported a patient who had received distal gastrectomy and gastroenterostomy due to peptic ulcer about 40 years ago who then presented with remnant gastric cancer along with a tumor thrombus in the splenic vein extending into the portal vein. However, to the best of our knowledge, there are no previous reports in the literature of a patient with tumor thrombus in the SMV via direct jejunal venous drainage.

Portal venous tumor thrombus in patients with gastric cancer, with or without liver metastasis, is a poor prognostic factor, and the survival of these patients is dismal [2, 3, 7, 11]. The overall survival is reported to be no longer than 6 months in such cases [2]. Although not based on literature data, it is thought that isolated tumor thrombus in the SMV might also be associated with poor prognosis. As a matter of fact, our case developed multiple liver metastases and peritoneal involvement within 22 months of follow-up.

Eom et al. retrospectively evaluated 51 gastric cancer patients with portal tumor thrombosis and reported that this patient group had a very poor prognosis with an average survival of 5.4 months [2]. In 2016, Sato et al. reviewed 71 cases of gastric cancer with portal vein thrombosis who received 2 to 4 cycles of S-1 and cisplatin therapy in Japan [3]. They reported 10 patients with a survival over 36 months in their study. Because splenic vein and SMV both drain into the portal system, the presence of a tumor thrombus in these vascular structures is also accepted as a poor prognostic factor and the life expectancy is very short in these patients.

## 4. Conclusion

It should be kept in mind that gastric cancer may lead to portal vein tumor thrombus or that it may rarely be associated with an isolated SMV tumor thrombus. The presence of SMV tumor thrombus indicates a poor prognosis. Tumor thrombosis in the SMV might be expected in patients with gastric cancer who had previously undergone partial gastrectomy, due to alterations in venous drainage routes.

## References

[1] Tanaka A., Takeda R., Mukaihara S. (2002). Tumor thrombi in the portal vein system originating from gastrointestinal tract cancer. *Journal of Gastroenterology*.

[2] Eom B. W., Lee J. H., Lee J. S. (2012). Survival analysis of gastric cancer patients with tumor thrombus in the portal vein. *Journal of Surgical Oncology*.

[3] Sato S., Nagai E., Taki Y. (2016). A long-surviving case of gastric cancer with main portal vein tumor thrombus after surgical resection and postoperative S-1 therapy. *Clinical Journal of Gastroenterology*.

[4] Furui J., Enjyoji A., Okudaira S., Takayama K., Kanematsu T. (1998). Successful surgical treatment of gastric cancer with a tumor thrombus in the portal and splenic veins: report of a case. *Surgery Today*.

[5] Viadana E., Bross I. D., Pickren J. W. (1978). The metastatic spread of cancers of the digestive system in man. *Oncology*.

[6] Sugawara Y., Konishi T., Hiraishi M. (1996). Portal tumor thrombi due to gastric cancer. *Hepatogastroenterology*.

[7] Ishigami S., Arigami T., Okubo K. (2012). Successful treatment of advanced gastric adenocarcinoma with portal tumor thrombosis by total gastrectomy following CDDP and S-1 therapy. *Clinical Journal of Gastroenterology*.

[8] Morishima K., Hosoya Y., Kurashina K. (2010). A case of gastric cancer with portal tumor thrombus successfully treated by surgical resection. *Journal of Japan Surgical Association*.

[9] Araki T., Suda K., Sekikawa T., Ishii Y., Hihara T., Kachi K. (1990). Portal venous thrombosis associated with gastric adenocarcinoma. *Radiology*.

[10] Ishikawa M., Koyama S., Ikegami T., Fukutomi H., Gohongi T. (1995). Venous tumor thrombosis and cavernous transformation of the portal vein in a patient with gastric carcinoma. *Journal of Gastroenterology*.

[11] Nakao S., Nakata B., Tendo M. (2015). Salvage surgery after chemotherapy with S-1 plus cisplatin for a-fetoprotein-producing gastric cancer with a portal vein tumor thrombus: a case report. *BMC Surgery*.

